# High prevalence and associated factors of red blood cell folate deficiency among first-trimester pregnant women in a neural tube defect high-risk Region of Tigray, Northern Ethiopia

**DOI:** 10.1371/journal.pone.0356720

**Published:** 2026-08-20

**Authors:** Birhane Alem Berihu, Hayelom Kebede Mekonen, Afework Mulugeta, Tony Magana, Yibrah Berhe Zelelow, Masresha Tessema, Feyissa Challa, Yohannes Belay, Mehari Meles, Wossene Habtu, Tigist Getahun, Yosef Tolcha, Tafere Gebreegziabher Belay

**Affiliations:** 1 Department of Anatomy, Faculty of Biomedical Sciences, College of Health Sciences, Mekelle University - Mekelle, Ethiopia; 2 Department of Nutrition, Faculty of Public Health, College of Health Sciences, Mekelle University - Mekelle, Ethiopia; 3 Department of Neurosurgery, Faculty of Medicine, College of Health Sciences, Mekelle University - Mekelle, Ethiopia; 4 Department of Obstetrics and Gynecology, Faculty of medicine, College of Health Sciences, Mekelle University - Mekelle, Ethiopia; 5 Ethiopian public health institute- Addis Ababa, Ethiopia; 6 Central Washington University, Department of Health Sciences, Ellensburg, United States of America; 7 Department of Pain and Neural Sciences, School of Dentistry, University of Maryland Baltimore, United States of America; University of Oulu: Oulun Yliopisto, FINLAND

## Abstract

**Background:**

The Tigray region of northern Ethiopia has one of the highest reported prevalences of neural tube defects (NTDs) globally. Red blood cell (RBC) folate is the gold-standard biomarker for assessing long-term folate status and predicting the risk of folate-sensitive NTDs. This study assessed RBC folate concentrations and dietary diversity among first-trimester pregnant women in Tigray.

**Methods:**

A facility-based cross-sectional study was conducted from March to April 2025 in five randomly selected public hospitals in Tigray, Ethiopia. A total of 400 first-trimester pregnant women (≤13 weeks of gestation) were selected using systematic random sampling. Sociodemographic, anthropometric, obstetric, and dietary data were collected using interviewer-administered questionnaires and a 24-hour dietary recall based on the Food and Agriculture Organization Minimum Dietary Diversity for Women (MDD-W) tool. RBC folate concentrations were measured using electrochemiluminescence immunoassay. Descriptive statistics summarized the data, and an exploratory L_2_-penalized (Ridge) logistic regression model was fitted because of the rarity of folate sufficiency.

**Results:**

The mean RBC folate concentration was 221.6 ± 68.6 ng/mL, with a median of 204.9 ng/mL. Overall, 98.8% of participants (395/400; 95% CI: 97.1%–99.6%) had RBC folate concentrations below the WHO-recommended threshold of 400 ng/mL for optimal NTD prevention. Additionally, 79.5% (318/400) of participants did not achieve the minimum dietary diversity threshold (<5 food groups). Among women who achieved minimum dietary diversity, 97.6% (80/82) remained folate deficient. In the exploratory Ridge regression, achieving minimum dietary diversity was positively associated with folate sufficiency (AOR = 1.82), whereas increasing maternal age was inversely associated (AOR = 0.67).

**Conclusions:**

This study demonstrates that maternal RBC folate deficiency was present in almost all first-trimester pregnant women in Tigray, highlighting a substantial public health concern in a region with a high burden of neural tube defects. The finding that clinical deficiency crosses all demographic and dietary tiers indicates that individual-level dietary modification and sporadic supplementation are insufficient to protect maternal reserves. Safely addressing this structural micronutrient emergency and reducing the high burden of NTDs requires an immediate transition toward mandatory, large-scale food fortification of staple grains in Northern Ethiopia.

## Background

Folate, a B-vitamin essential for DNA synthesis, repair, and methylation, plays a critical role in cellular division and growth, particularly during periods of rapid fetal development in early pregnancy [1–3]. Inadequate maternal folate levels during the periconceptional period have been strongly associated with neural tube defects (NTDs) a group of severe congenital malformations of the brain and spinal cord, including spina bifida and anencephaly [3,4]. NTDs are among the most common congenital anomalies worldwide and contribute substantially to neonatal mortality, accounting for approximately 300,000 neonatal deaths annually [5,6]. Red blood cell (RBC) folate is considered the most reliable biomarker for assessing long-term folate status and correlates strongly with NTD risk [1,2,4]. Unlike serum folate, which reflects short-term status, RBC folate indicates longer-term folate stores. The World Health Organization (WHO) recommends RBC folate concentrations ≥400 ng/mL to minimize the risk of NTDs [7–9]. Despite global efforts to promote folic acid supplementation and food fortification, folate deficiency remains a public health challenge, especially in low-resource settings [10–12].

In many sub-Saharan African countries, particularly in rural and underserved regions, folate deficiency is widespread due to poor dietary diversity, limited access to fortified foods, and low awareness of maternal nutrition [13–16]. Persistent structural and systemic barriers continue to undermine the effectiveness of global folate supplementation efforts, leaving many women of reproductive age in Africa at elevated risk of folate deficiency and consequently, of giving birth to infants with NTDs. Ethiopia has one of the highest reported NTD burdens globally estimated at 71.48 per 10,000 births—ten times higher than the 2030 Sustainable Development Goal (SDG) target [17]. Among all the regions in Ethiopia, Tigray presents the most alarming figures, with NTD prevalence ranging from 130 to 262 per 10,000 births [18–20]. This high burden indicates a critical gap in maternal nutrition, particularly concerning folate status, during the early stages of pregnancy. Maternal folate status during the first 28 days post-conception is especially vital, as this is the window when neural tube closure occurs. However, there is a paucity of data on RBC folate levels among pregnant women in high-risk areas such as Tigray, Ethiopia. Therefore, this study aimed to address this gap by evaluating RBC folate concentrations among first-trimester pregnant women in the Tigray region of Ethiopia. It will also assess their nutritional and demographic profiles. The findings are expected to inform evidence-based public health policies and interventions aimed at preventing NTDs through high impact maternal nutrition interventions.

## Methodology

### Study setting and period

The study was conducted in Tigray, identified as a high-risk setting for NTDs, located in northern Ethiopia. Moreover, Tigray is one of the twelve administrative regions and two administrative cities in Ethiopia. Administratively, Tigray is divided into seven zones, namely Central, Eastern, Mekelle, North Western, South Eastern, Southern, and Western Zones. The region is further divided into 94 districts (locally called woredas). In the Tigray region, there are approximately 992,635 households, with an average household size of 3.4 persons in urban areas and 4.6 in rural areas [21]. Socioeconomic indicators reveal that 31.6% of the population falls within the lowest wealth quintile. Adult literacy rates show a significant gender gap, with 67.5% of men and only 33.7% of women being literate [22]. According to the 2016 Ethiopian Demographic and Health Survey (EDHS), antenatal care (ANC) coverage for at least one visit (ANC1) was 90%, while coverage for four or more visits (ANC4+) stood at 57%. Additionally, 56.9% of births in the region were reported to have occurred in health facilities, reflecting a moderate level of institutional delivery service utilization [23]. Apart from the private health facilities, the health system in Tigray includes 741 village health posts, 230 health centers, 24 primary hospitals, 14 general hospitals, and two referral (tertiary) hospitals. Data were collected from one tertiary hospital, one general hospital, and three primary hospitals. These were Ayder Comprehensive Specialized Hospital (tertiary level), Lemlem Karl General Hospital, Adishu Primary Hospital, Samre Primary Hospital, and Yechila Primary Hospital from March to April 2025. These hospitals provide 24-hour obstetrics and gynecology care and different type of health care services for their representative population.

### Study design

Cross-sectional laboratory-based study was conducted in selected health institutions across Tigray, northern Ethiopia.

### Sample size

The required sample size was determined using a single-population proportion formula, assuming a 49% prevalence of folate deficiency among pregnant women in Haramaya District, Eastern Ethiopia [30]. With a 95% confidence level and a 5% margin of error, the initial calculated sample size was 384. To account for a potential 10% non-response rate, the final required sample size was increased to 422 participants.

### Sampling technique

The five participating public hospitals (one tertiary, one general, and three primary) were selected from an eligible pool of government facilities across accessible zones in Tigray using a computer-generated random number sequence. The baseline sampling frame comprised all first-trimester pregnant women booked for antenatal care (ANC) visits during the March–April 2025 study period. Based on ANC registration logs, the total number of eligible women across the five hospitals was 1,240: Ayder Comprehensive Specialized Hospital (n = 155), Lemlem Karl General Hospital (n = 465), Adishu Primary Hospital (n = 155), Samre Primary Hospital (n = 310), and Yechila Primary Hospital (n = 155). The final required sample size (n = 422) was proportionally allocated to each hospital according to its share of eligible women using the largest-remainder method: Ayder n = 53, Lemlem Karl n = 158, Adishu n = 53, Samre n = 105, and Yechila n = 53 (summing to 422). Within each hospital, participants were selected using systematic random sampling. The sampling interval (k) was calculated separately for each hospital by dividing the total eligible women by the allocated sample (e.g., for Ayder: 155/53 ≈ 3).

A random starting point between 1 and 3 was selected by lottery at each hospital, after which every 3rd eligible woman listed on the ANC register was invited until that hospital’s allocation was attained. If the end of a clinic day’s register was reached before meeting the allocation, recruitment continued on subsequent clinic days using the same interval and procedure. Eligibility was verified from ANC logs at the point of approach; duplicate entries (e.g., repeat visits) were removed using unique ANC identifiers to avoid resampling the same individual. Non-responders were not replaced; reasons for non-participation were recorded. In total, 422 women were approached; 400 consented and completed the study (response rate: 94.8%), with non-participation due to refusal (n = 22).

### Study population

The study population included pregnant women in their first trimester (≤13 weeks gestation as confirmed by ultrasound and last menstrual period) during their antenatal care follow up.

### Eligibility criteria

Inclusion criteria: Pregnant women in their first trimester (≤13 weeks of gestation), confirmed by ultrasound examination and last menstrual period (LMP), were eligible for inclusion. Participants were required to have been permanent residents of the Tigray region for at least six consecutive months before enrollment. Residency was verified using local administrative registration records to ensure that the study population was representative of the regional catchment area and to minimize inclusion of women referred from outside the study area.

Exclusion criteria: Women were excluded if they had:

- Hematological disorders or chronic diseases (e.g., HIV/AIDS, diabetes)
- Current or recent malaria infection or use of antimalarial drugs
- History of folic acid supplementation before conception
- Alcohol or substance abuse, as it impairs folate metabolism
- Twin pregnancies alter folic acid metabolism differently than singleton pregnancies
- MTHFR gene mutations (if known or reported by the mother)

### Study variables

Dependent variable: RBC folate concentration (ng/mL)

Independent variables: socio-demographic and economic status, obstetric history, health-related behaviors, and nutritional status.

### Data collection procedures

#### Sociodemographic.

Trained data collectors used a structured and pretested questionnaire to collect information on sociodemographic characteristics such as age, marital status, residence, education, occupation, perceived economic status; Obstetric history such as parity, pregnancy planning, previous outcomes; health-related behaviors such as health care service utilization, and family support.

### Dietary Diversity, Food Frequency, and Anthropometric Assessment

Dietary intake was assessed during the participants’ first antenatal care (ANC) visit using a 24-hour food group recall and a structured Food Frequency Questionnaire (FFQ) administered through face-to-face interviews. The 24-hour food recall was used to compute individual Dietary Diversity Scores (DDS) and assess Minimum Dietary Diversity for Women (MDD-W). Foods consumed during the preceding 24 hours were classified into the 10 FAO food groups according to the Food and Agriculture Organization (FAO) MDD-W guideline. MDD-W attainment was defined dichotomously as achieved (consumption of ≥5 food groups) or not achieved (consumption of <5 food groups), in accordance with FAO recommendations [24]. The FFQ was used to capture the frequency of consumption of the major food groups during the same 24-hour recall period, thereby complementing the dietary diversity assessment.

Anthropometric assessment was performed following standard WHO procedures. Mid-upper arm circumference (MUAC) was measured on the midpoint of the left upper arm using a non-stretchable measuring tape while participants were seated comfortably with the arm relaxed. Measurements were recorded to the nearest 0.1 cm. Maternal nutritional status was classified according to WHO recommendations, with MUAC <23 cm indicating maternal undernutrition and MUAC ≥23 cm indicating normal nutritional status.

### Laboratory investigation

#### Sample collection and handling.

Approximately 4 mL of venous blood was collected aseptically into EDTA vacutainer tubes from each participant during their antenatal care visit. The samples were labeled and immediately stored at 2–8°C in portable cold boxes at the collection sites in Tigray, Ethiopia.

### Sample transportation and storage

The collected sample were stored at –20°C in deep freezer at each collection sites until transported to EPHI for analysis. Blood samples were transported in cold-chain conditions (2–8°C) from the health facilities in Tigray to the Ethiopian Public Health Institute (EPHI) in Addis Ababa. Upon arrival at EPHI, all samples were stored at –20°C until analysis. Samples were processed within three weeks from the date of collection, in accordance with manufacturer and WHO recommendations for RBC folate stability.

### Preparation of the hemolysate sample

RBC folate was measured using the Elecsys® Folate RBC assay (Test No. 1210) on the Cobas e 411 analyzer (Roche Diagnostics GmbH, Mannheim, Germany), based on electrochemiluminescence immunoassay (ECLIA) technology. Sample preparation followed Roche’s standard protocol as described below. 100 μL of whole blood was mixed with 3.0 mL of Folate RBC hemolyzing reagent (0.2% ascorbic acid solution). The hemolysate was incubated at 20–25°C for 90 ± 15 minutes with caps closed. The samples were stored at –20°C (±5°C) and analyzed within one month allowing only one freeze-thaw cycle.

### Laboratory analysis of RBC folate

Red blood cell (RBC) folate concentrations were measured using the Elecsys® Folate RBC assay (Test No. 1210) on the Cobas e 411 analyzer (Roche Diagnostics GmbH, Mannheim, Germany), a fully automated platform based on electrochemiluminescence immunoassay (ECLIA) technology.

### Assay Principle

The Cobas e 411 system uses a competitive binding principle based on electrochemiluminescence immunoassay (ECLIA). In the first step, the folate in the sample competes with a ruthenium-labeled folate derivative for binding sites on the folate-binding protein (FBP) immobilized on the solid phase (streptavidin-coated microparticles). After washing to remove unbound substances, a voltage is applied to the reaction mixture, triggering a light-emitting reaction. The chemiluminescent signal produced by the bound ruthenium complex is inversely proportional to the amount of folate in the sample.

### Calibration and quality control

Blood samples were processed and analyzed for RBC folate levels at the Ethiopian Public Health Institute (EPHI) using the Elecsys® Folate RBC assay on the Cobas e 411 automated platform. To verify analytical precision, two distinct levels of quality control materials (Folate RBC Control 1 and 2) were run alongside each patient batch. The assay demonstrated optimal stability and reproducibility, with an intra-assay coefficient of variation (CV) between 3.2% and 4.5% and an inter-assay CV below 6.0%.

### Calculation of RBC folate concentration

RBC folate concentration was calculated using the following formula.


$$\begin{array}{c} RBC\;Folate\;(ng/mL)=\frac{Total\;Folate\;in\;Hemolysate\;(ng/mL)\times Dilution\;Factor}{Hematocrit} \end{array}$$

Hematocrit values were obtained from complete blood count (CBC) analysis conducted in parallel, and the final results were expressed in ng/mL. Folate status was determined based on the WHO-recommended cutoff value: < 400 ng/mL: folate deficiency (associated with increased NTD risk) or ≥ 400 ng/mL: folate sufficiency.

### Biosafety

All laboratory activities were conducted at the Ethiopian Public Health Institute under strict biosafety protocols in accordance with standard operating procedures (SOPs) and Good Laboratory Practice (GLP) guidelines. Biosafety procedures followed the EPHI laboratory biosafety manual. All personnel were trained in sample handling, and equipment maintenance was performed regularly.

#### Statistical Analysis.

Data were entered and cleaned using IBM SPSS Statistics version 27.0 (IBM Corp., Armonk, NY, USA), and statistical analyses were performed using SPSS and Python (scikit-learn). Continuous variables were assessed for normality using the Shapiro–Wilk test. Because RBC folate concentrations were not normally distributed, continuous variables are summarized as means ± standard deviations (SD), medians with interquartile ranges (IQR), and ranges, whereas categorical variables are presented as frequencies and percentages. Maternal RBC folate status was classified according to the World Health Organization (WHO) threshold for neural tube defect prevention as deficient (<400 ng/mL) or sufficient (≥400 ng/mL). Minimum Dietary Diversity for Women (MDD-W) was classified according to FAO guidelines as achieved (≥5 food groups) or not achieved (<5 food groups).

Because only five participants (1.3%) had sufficient RBC folate concentrations, resulting in extreme class imbalance, associations between categorical variables and RBC folate status were assessed using Fisher’s exact test or the Fisher–Freeman–Halton exact test, as appropriate. Multivariable analysis was performed using L_2_-penalized (Ridge) logistic regression to reduce coefficient instability associated with sparse data. Predictor variables included maternal age, gestational age, MDD-W status, MUAC, residence, and perceived socioeconomic status. Continuous variables were standardized before model fitting, and adjusted odds ratios (aORs) with 95% confidence intervals (CIs) were reported.

Model performance was evaluated using stratified 5-fold cross-validation. Model discrimination was assessed using the area under the receiver operating characteristic curve (AUC-ROC) and the precision–recall area under the curve (PR-AUC), while calibration was evaluated using the Brier score. Sensitivity analyses were conducted using both the original dataset and a dataset balanced through minority-class upsampling performed within each cross-validation fold to minimize data leakage. Because of the extremely small number of folate-sufficient participants, the regression and validation analyses are presented as exploratory, and the study’s primary conclusions are based on the descriptive prevalence estimates. Statistical significance was defined as a two-sided p < 0.05.

### Ethical Considerations

Ethical Approval and Consent to Participate: This protocol was reviewed and approved by the Institutional Review Board of the College of Health Sciences, Mekelle University (Approval No. MU-IRB 2411/2024) and conducted in strict accordance with the Declaration of Helsinki. Orally informed consent was obtained from all participants prior to enrollment. The use of an oral consent workflow was explicitly approved by the IRB to account for varying literacy profiles within the target demographic and to minimize participant anxiety regarding formal documentation during routine clinical workflows.

## Results

### Sociodemographic characteristics

Table 1 summarizes the sociodemographic and obstetric characteristics of the participants. The mean maternal age was 24.78 years (SD ± 5.04), with a median of 24.0 years (range: 15–38). More than half of the participants (55.0%) resided in urban areas, while the remainder lived in rural settings. A substantial majority (71.0%) perceived themselves as economically poor, highlighting socioeconomic barriers that may impact maternal nutrition and access to healthcare services.

**Table 1:** Sociodemographic characteristics of first trimester pregnant women in a neural tube defect high-risk area of Tigray, Ethiopia.

Characteristics		Frequency	Percent
Maternal age	15-19	57	14.2
	20-24	153	38.3
	25-29	105	26.3
	30-34	63	15.8
	>=35	22	5.5
Marital status	Married	373	93.3
	unmarried	22	5.5
	Divorced	5	1.3
Residence	Rural	180	45.0
	Urban	220	55.0
Educational status	No formal education	37	9.3
	Elementary school	137	34.3
	Highschool	178	44.5
	College diploma and above	48	12.0
Occupation	House wife	299	74.8
	Labor	14	3.5
	Student	22	5.5
	Merchant	48	12.0
	College educated professional	17	4.3
Assumed economic status	Poor	284	71.0
	Medium	105	26.3
	Rich	11	2.8

Maternal age: mean 24.78 ± 5.04 years; median 24.0 years (range 15–38). Standard Deviation (SD): 5.04 years.

The occupational profile showed that 74.8% of participants were housewives, reflecting a high level of economic dependence. Educational attainment was relatively low. Only 12.0% had achieved a college diploma or higher. Most participants had completed high school (44.5%) or elementary school (34.3%), and 9.3% reported having no formal education. These educational patterns suggest potential limitations in health literacy, which could influence dietary habits and healthcare-seeking behaviors.

### Obstetric characteristics

Healthcare engagement was suboptimal. About 51.0% of women reported rare or limited use of healthcare services, and only 28.5% attended regular antenatal check-ups. Similarly, only 28.5% reported receiving family support for healthcare utilization, indicating a lack of strong social support systems, which may adversely affect both maternal and fetal outcomes. In terms of obstetric history, 52.5% of participants were multiparous, while 47.5% were nulliparous. Among those with previous pregnancies, 50.0% had experienced full-term live births, 1.8% had a history of stillbirth, miscarriage, or abortion, and 0.8% had preterm births. Notably, 86.8% of current pregnancies were unplanned, indicating considerable gaps in reproductive health education and access to family planning services (Table 2).

**Table 2:** Obstetric characteristics of first-trimester pregnant women in a neural tube defect high-risk area of Tigray, Ethiopia.

Characteristics		Frequency	Percent
Health care service utilization	Occasional (Visits as needed)	82	20.5
	Rare (Limited visits)	204	51.0
	Regular (Frequent check-ups)	114	28.5
Family support for health care use	No	286	71.5
	Yes	114	28.5
Parity	Multipara (Has given birth before)	210	52.5
	Nullipara (First-time mother)	190	47.5
Use planned pregnancy	No	347	86.8
	Yes	53	13.3
Pregnancy outcome before the current pregnancy	Preterm live birth	3	.8
	Stillbirth/Miscarriage/Abortion	7	1.8
	First trimester pregnant mother	190	47.5
	Full-term live birth	200	50.0
Sex or previous pregnancy outcome	unknown	4	1.0
	Male	105	26.3
	Female	101	25.3
	First trimester pregnant mother	190	47.5

### Clinical and nutritional characteristics

At the time of data collection, the majority of participants (60.3%) were between the 9th and 12th weeks of gestation, 31.8% were between the 5th and 8th weeks, and 8.0% were in the first four weeks. This distribution reflects the study’s focus on assessing folate levels during the critical period of neural tube formation (Table 3). Nutritional status assessments revealed that over half of the participants (56.0%) had a MUAC below 23 cm, while 44.0% had a MUAC of 23 cm or higher (Table 3).

**Table 3:** Clinical and nutritional characteristics of first trimester pregnant women in a neural tube defect high-risk area of Tigray, Ethiopia.

Characteristics		Frequency	Percent
Gestational age	1-4 weeks	32	8.0
	5-8 weeks	127	31.8
	9-12 weeks	241	60.3
MUAC	Low MUAC (<23 cm)	224	56.0
	Normal MUAC (≥23 cm)	176	44.0

MUAC: mean 21.75 ± 1.86 cm; median 22.0 cm (range 17.0–26.0). Standard Deviation (SD): 1.86 cm

### Dietary patterns of the study participants

The analysis of food group consumption among participants reveals significant insights into dietary diversity within the study population. A striking 99.25% of respondents reported consuming grains, white roots and tubers, and plantains, indicating that these staples form the foundation of the typical diet. Pulses such as beans, peas, and lentils were consumed by 66.75% of participants, suggesting a moderate inclusion of plant-based protein sources.

Consumption of nutrient-dense food groups such as nuts and seeds (26.25%), dark green leafy vegetables (39.25%), and other vitamin A-rich fruits and vegetables (27.00%) was comparatively lower, pointing to potential gaps in micronutrient intake. Alarmingly, the intake of animal-source foods was particularly limited: only 9.75% consumed dairy, 12.50% consumed meat, poultry, or fish, and 12.00% consumed eggs. (Table 4).

**Table 4:** Frequency and percentage of food group among the participant mothers consumed in 24 hours dietary recalls period.

Food Group	Count	% of Respondents
Grains, white roots and tubers, and plantains	397	99.25%
Pulses (beans, peas and lentils)	267	66.75%
Nuts and seeds	105	26.25%
Dairy	39	9.75%
Meat, poultry and fish	50	12.50%
Eggs	48	12.00%
Dark green leafy vegetables	157	39.25%
Other vitamin A-rich fruits and vegetables	108	27.00%
Other vegetables	67	16.75%
Other fruits	67	16.75%

The distribution of Dietary Diversity Scores (DDS), based on the FAO’s 10-food group classification, reveals a concerning trend in the dietary patterns of the study population. The analysis revealed that an overwhelming majority of the first-trimester mothers (79.5%, n = 318) failed to achieve the minimum target of consuming ≥5 separate food groups within the 24-hour assessment period (Table 5). These figures reflect a limited inclusion of nutritionally important food groups such as fruits, vegetables, dairy, and animal-source proteins, corroborating the low intake patterns observed in the food group frequency analysis.

**Table 5:** The distribution of Dietary Diversity Scores (DDS), based on the FAO’s 10-food group classification.

MDD-W Attainment Status	Dietary Characteristic	Total Cohort (N = 400)
Not Achieved	<5 Food Groups	318 (79.5%)
Achieved	≥5 Food Groups	82 (20.5%)

### RBC Folate Status of First-Trimester Pregnant Women in a Neural Tube Defect High-Risk Area of Tigray, Ethiopia (n = 400)

**Fig 1:**
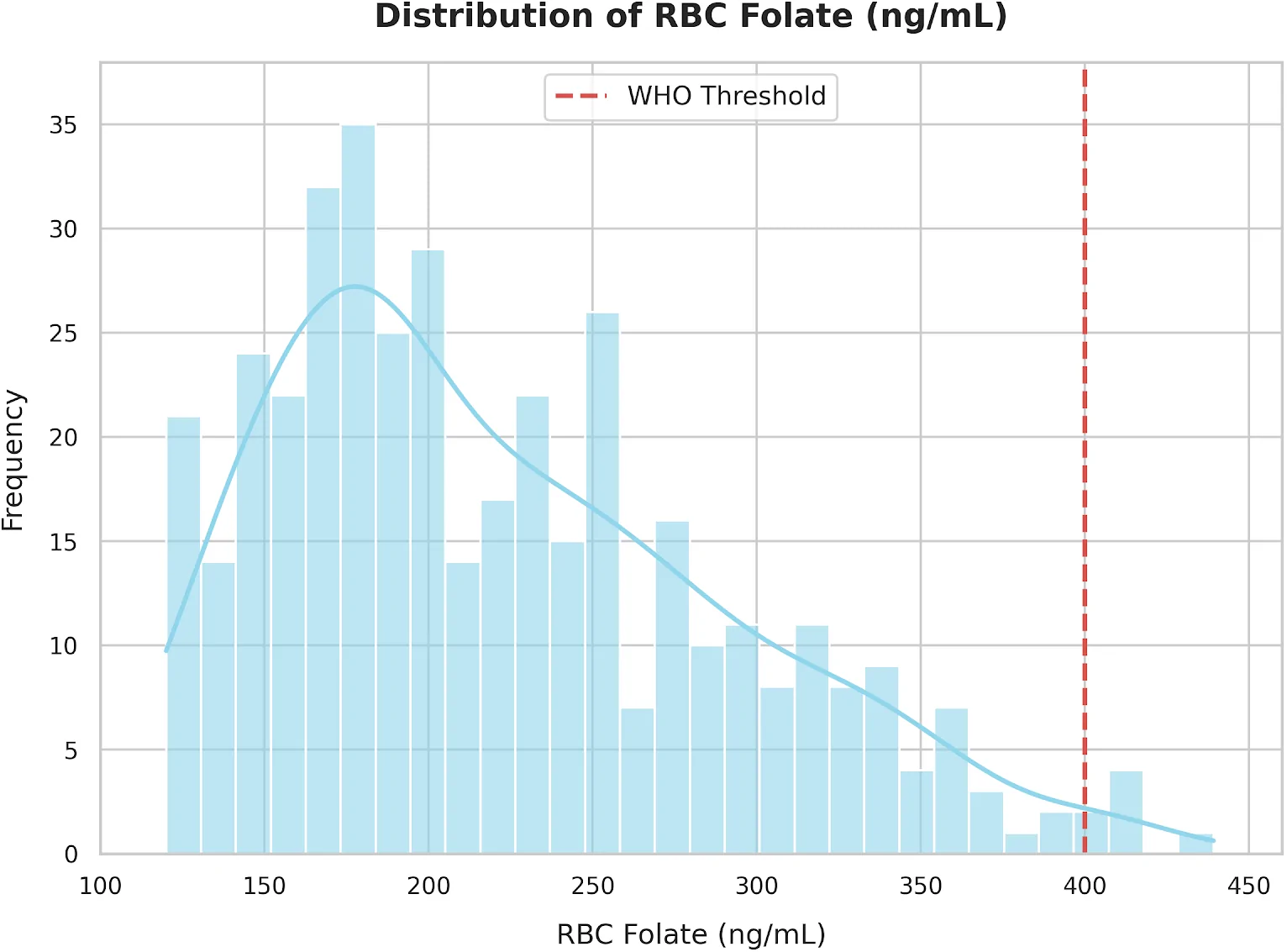
Red Blood Cell (RBC) folate distribution across the cohort against the WHO preventive threshold.

Table 6 summarizes the distribution of RBC folate concentrations among the study participants. Overall, 395 of the 400 participants (98.8%; 95% CI: 97.1%–99.6%) had RBC folate concentrations below the WHO-recommended threshold of 400 ng/mL for optimal neural tube defect prevention, whereas only 5 participants (1.3%) had concentrations ≥400 ng/mL (Table 6 and Fig 1). The mean RBC folate concentration was 221.6 ± 68.6 ng/mL, with a median of 204.9 ng/mL, indicating generally low folate status. The Shapiro–Wilk test demonstrated that RBC folate concentrations were not normally distributed (W = 0.929, p < 0.0001).

**Table 6:** Descriptive RBC Folate Status of First-Trimester Pregnant Women in a Neural Tube Defect High-Risk Area of Tigray, Ethiopia (n = 400).

Clinical Biomarker	Value/ Metric
RBC folate deficiency prevalence, below the WHO threshold (<400 ng/mL) for NTD prevention.	98.8% (n=395)
RBC folate sufficiency prevalence, meeting the WHO threshold (≥400 ng/mL).	1.25% (n=5)
Mean RBC Folate Concentration	221.6±68.6 ng/mL
Median RBC Folate Concentration	204.9 ng/mL
Range (Minimum – Maximum)	120.0−439.3 ng/mL
Shapiro-Wilk Normality Test	W=0.929,p<0.0001

### Distribution of maternal RBC folate status categorized by the FAO Minimum Dietary Diversity for Women (MDD-W)

When cross-tabulated with biological outcomes, 99.1% (n=315) of the women who failed to meet the MDD-W thresholds were simultaneously diagnosed with clinical RBC folate deficiency (Table 7). Interestingly, even among the minority who achieved the dietary target (n=82), 97.6% (n=80) still presented with deficient blood profiles. This widespread cross-over effect resulted in a non-significant exact p-value (p=0.273), illustrating that systemic, regional micronutrient depletion in the local food supply is so pervasive that meeting general dietary group volume targets alone is insufficient to protect maternal red blood cell folate reserves in this region.

**Table 7:** Distribution of maternal RBC folate status categorized by the FAO Minimum Dietary Diversity for Women (MDD-W) indicator (N=400).

		RBC folate status		Total Cohort (N = 400)	p-value)
**MDD-W Attainment Status**	**Dietary Characteristic**	**Deficient (n = 395)**	**Sufficient (n = 5)**
Not Achieved	<5 Food Groups	315	3	318 (79.5%)	0.273 (Fisher’s Exact Test)
Achieved	≥5 Food Groups	80	2	82 (20.5%)	

### Bivariate Analysis of Dietary Diversity and Maternal Characteristics

Table 8 presents the unadjusted bivariate associations between maternal characteristics and RBC folate status based on the original, unmanipulated dataset. Overall, 79.5% (318/400) of participants did not achieve the FAO Minimum Dietary Diversity for Women (MDD-W) threshold (<5 food groups). Among these women, 99.1% (315/318) had RBC folate concentrations below the WHO-recommended threshold of 400 ng/mL. Likewise, 97.6% (80/82) of women who achieved minimum dietary diversity (≥5 food groups) were also folate deficient. The association between MDD-W attainment and RBC folate status was not statistically significant (p = 0.273).

**Table 8:** Bivariate Association of Dietary and Anthropometric Factors with RBC Folate Status (N=400).

Predictor Variable	Category	RBC folate status		Total (N = 400)	p-value)
**Deficient (n = 395)**	**Sufficient (n = 5)**
Dietary Diversity *(FAO MDD-W Benchmark)*	Not Achieved (<5 groups)	315	3	318 (79.5%)	p=0.273 *(Fisher’s Exact)*
	Achieved (≥5 groups)	80	2	82 (20.5%)	
Maternal Age	15–19 years	35	0	35 (8.8%)	p=0.918 *(Chi-Square)*
	20–24 years	147	2	149 (37.3%)	
	25–29 years	119	2	121 (30.3%)	
	30–34 years	69	1	70 (17.5%)	
	35–39 years	25	0	25 (6.3%)	
Nutritional Wasting Status *(Maternal MUAC)*	Low (<23 cm)	220	4	224 (56.0%)	p=0.390 *(Fisher’s Exact)*
	Normal (≥23 cm)	175	1	176 (44.0%)	

Similarly, no significant associations were observed between RBC folate status and maternal age group (p = 0.918) or nutritional status assessed by mid-upper arm circumference (MUAC) (p = 0.390). Although 56.0% (224/400) of participants had MUAC <23 cm, indicating maternal undernutrition, the prevalence of RBC folate deficiency remained consistently high across all age, dietary diversity, and nutritional status categories. These findings suggest that RBC folate deficiency was nearly universal within the study population, regardless of individual demographic or nutritional characteristics.

### Factors Associated with RBC Folate Status among first trimester pregnant women (Penalized Ridge Regression)

To address multicollinearity and the extreme class imbalance (395 folate-deficient vs. 5 folate-sufficient participants), an exploratory L_2_-penalized (Ridge) logistic regression model was fitted (Table 9). After regularization, achieving the FAO Minimum Dietary Diversity for Women (MDD-W) threshold (≥5 food groups) was associated with higher odds of folate sufficiency (AOR = 1.82), whereas increasing maternal age showed an inverse association (AOR = 0.67). Rural residence (AOR = 2.13) and MUAC <23 cm (AOR = 2.62) was also associated with higher estimated odds of folate sufficiency. However, these estimates should be interpreted with considerable caution because only five participants met the WHO threshold for RBC folate sufficiency. Consequently, the regression analysis is presented as exploratory, and the study’s primary conclusions are based on the descriptive prevalence estimates rather than the model coefficients.

**Table 9:** Factors associated with RBC folate sufficiency among first-trimester pregnant women via regularized penalized logistic regression ($L_{2}$ Ridge Penalty).

Predictor Variable	Standardized Coefficient (β)	Adjusted Odds Ratio (AOR)	Directional Association and Context
MDD-W Achievement (Achieved ≥5 groups versus Not)	+0.599	1.820	Positive association: Meeting dietary thresholds tracks with an 82% increase in the odds of adequate folate status.
Nutritional Wasting (MUAC) (Low <23 cm versus Normal)	+0.963	2.621	Reflects highly concentrated sampling parameters due to the low absolute event count (n=5 sufficient).
Residential Locality (Rural versus Urban)	+0.758	2.133	Points toward localized geographic clustering of the preserved profiles.
Socioeconomic status (Poor versus Medium/Rich)	+0.173	1.188	Minimal independent directional contribution when controlling for dietary metrics.
Maternal Age (Continuous)	−0.404	0.668	Inverse association: Advancing maternal age correlates with a gradual decrease in relative odds of adequacy.
Gestational Age (Weeks)	+0.120	1.127	Minimal structural variation across the narrow first-trimester clinical boundary.

### Predictive Performance and Validation Metrics for RBC Folate Sufficiency Classification

Model performance was evaluated using stratified 5-fold cross-validation to preserve the distribution of the rare outcome across validation folds (Table 10). Model discrimination was assessed using the area under the receiver operating characteristic curve (AUC-ROC) and the precision–recall area under the curve (PR-AUC), while calibration was evaluated using the Brier score. Because only five participants had RBC folate concentrations ≥400 ng/mL, validation metrics should be interpreted with caution. Results from models using minority-class upsampling were considered exploratory, as data replication does not generate new information or improve model generalizability.

**Table 10:** Predictive Performance and Validation Metrics for RBC Folate Sufficiency Classification.

Diagnostic/ Validation Metric	Original Unadjusted Model (N = 400)	Balanced Up sampled Model (Exploratory)	Practical Methodological Meaning and Validation Interpretation
Stratified Cross-Validation Accuracy	98.8%	68.5%−78.2%	The original model’s high accuracy is a mathematical artifact driven entirely by the 98.8% majority class prevalence.
Area Under the ROC Curve (AUC-ROC)	0.54	0.81	Upsampling builds an artificial mathematical trajectory, simulating high sensitivity by repeating identical patient vectors.
Precision-Recall AUC (PR-AUC)	0.02	0.53	Highlights the severe true positive limitations; the true baseline probability of capturing sufficiency is only 1.25%.
Brier Score (Calibration)	0.012	0.185	A lower original score indicates mechanical alignment with the dominant class, not authentic clinical prediction capacity.
Sensitivity (Recall for Sufficiency)	0.0%	80.0%	The unadjusted model labels all mothers “deficient” to maintain mechanical mathematical accuracy.
Specificity (Detection of Deficiency)	100.0%	65.4%	Balancing the dataset forces the model to sacrifice true specificity in order to capture the rare synthetic positive cases.

## Discussion

This study reveals an exceptionally high prevalence of red blood cell (RBC) folate deficiency among first-trimester pregnant women in a neural tube defect (NTD) high risk region of northern Ethiopia. Nearly all participants (98.8%) had RBC folate levels below the World Health Organization’s recommended threshold of 400 ng/mL for NTD prevention [8,9]. This widespread deficiency ranks among the highest reported globally and poses serious risks to both maternal and fetal health in the region. The disparity in folate deficiency rates between this study and findings from other regions of Ethiopia is notable. In Addis Ababa, a study using the same biomarker and threshold reported a 27% prevalence of RBC folate deficiency among first-trimester pregnant women [25]. Our finding (98.8%) is significantly higher than the 2015 national micronutrient survey found that 32% of women of reproductive age had RBC folate levels below the threshold for NTD prevention [26–28]. This emphasizes that Tigray requires a region-specific public health intervention rather than a one-size-fits-all national policy. A community-based study in eastern Ethiopia (Haramaya District), using serum folate rather than RBC folate, reported a 49% deficiency rate [29]. These variations may result from differences in biomarkers serum folate reflects short-term intake, while RBC folate indicates long-term status as well as regional disparities in diet, socioeconomic factors, healthcare access, and study design. Urban areas like Addis Ababa likely have better dietary diversity and access to folate-rich foods than rural regions like Tigray and Haramaya, contributing to the differences observed.

Assessing folate landscapes across sub-Saharan Africa reveals substantial variation that is frequently driven by differing laboratory biomarkers and cutoff parameters. For example, Côte d’Ivoire [30] documented a folate deficiency prevalence of 86.1% using the gold-standard RBC biomarker (cutoff <400 ng/mL). In contrast, studies utilizing serum folate—which captures transient, short-term intake rather than long-term cellular stores—show highly variable rates, such as 54.8% in Senegal [31] (cutoff <3 ng/mL), 18.8% in Benin [32], and 9% in Nigeria [33]. In Ethiopia, a study conducted among adolescents aged 15–19 found a 31.3% deficiency rate [34], while a national study across nine administrative regions reported 46% among women of reproductive age [35]. South Africa successfully eliminated clinical deficiency (0%) following the introduction of mandatory wheat and maize flour fortification [36]. In Cameroon, deficiency rates were 17% in women and 8% in children [37]. In Mbeya, Tanzania, 24% of pregnant women had serum folate levels below 3 ng/mL [38]. When contextualized against these continental findings, the 98.8% RBC folate deficiency rate observed in Tigray represents one of the highest documented burdens, reinforcing the need for region-specific public health responses.

International comparisons highlight the unusually severe nature of the problem in our study setting, underscoring its urgency and the need for targeted intervention. According to a global systematic review of folate status among women of reproductive age (WRA), folate deficiency (FD) affects over 20% of women in many low-income countries, far exceeding the threshold considered a public health concern. In contrast, the prevalence is typically below 5% in high-income countries [39]. Regional studies further illustrate this disparity: in Venezuela, [40] folate deficiency prevalence was reported at 36.3%, and in Turkey, it reached 71.7% among women [41]. These figures underscore the exceptional burden observed in this Ethiopian setting.

Our study has shown low DDS, medium DDS, residence, and age was found as the strongest risk factors for RBC folate deficiency among first-trimester pregnant women. our finding is consistent with findings from Addis Ababa and national studies that link micronutrient adequacy to diets rich in vegetables, legumes, and animal-source foods [25–28]. The findings carry serious public health implications. The widespread deficiency aligns with persistently high rates of NTDs reported in Tigray, ranging from 130 to 262 per 10,000 births [8–10], among the highest in sub-Saharan Africa. A critical contributing factor is the lack of pregnancy planning; over 86% of participants reported unplanned pregnancies, reducing the likelihood of periconceptional folic acid supplementation. This is consistent with national trends and highlights the urgent need to integrate micronutrient counselling into family planning and reproductive health services.

Mandatory food fortification presents a promising, evidence-based strategy to address widespread folate deficiency. Global meta-analyses have demonstrated that folic acid fortification of staple grains significantly reduces the incidence of NTDs [42]. Although Ethiopia has drafted fortification regulations, implementation remains pending. Our findings highlight the urgent need to accelerate this policy, particularly in high-risk regions such as Tigray. Meanwhile, promoting the consumption of locally available, folate-rich foods such as dark green leafy vegetables, legumes, and animal-source products is critical. Our finding has shown most respondents (99.25%) consumed staple foods, while intake of nutrient-rich and animal-source foods was low, indicating poor dietary diversity and potential nutrient gaps. The data suggest a heavy reliance on starchy staples and an underrepresentation of several key food groups, particularly those rich in protein and micronutrients. These findings highlight the need for targeted nutrition interventions to promote more balanced diets that include a greater variety of fruits, vegetables, and animal-source foods to improve overall nutritional status. A tailored food systems approach that improves access to and intake of folate-rich foods is urgently required to complement broader policy interventions.

## Conclusion

This study demonstrates that maternal RBC folate deficiency was present in almost all first-trimester pregnant women in Tigray, highlighting a substantial public health concern in a region with a high burden of neural tube defects. With 98.8% of the cohort exhibiting clinically significant red blood cell (RBC) folate deficiency and 79.5% failing to achieve the FAO Minimum Dietary Diversity for Women (MDD-W) threshold, the biochemical data confirm that the current nutritional status of these expectant mothers is critically inadequate to support early fetal development and prevent congenital anomalies.

Our multivariable analysis, utilizing $L_{\text{2}}$ penalized (Ridge) logistic regression to account for the extreme baseline class imbalance, reveals that while achieving minimum dietary diversity is a statistically significant modifiable factor for folate retention (AOR=1.82), it is insufficient to correct the systemic depletion observed across the study population. The near-universal nature of the deficiency, coupled with the lack of significant protective effects from other demographic and anthropometric variables, suggests that the folate crisis in this region is structural rather than behavioral. Individual-level dietary advice, while necessary, cannot overcome a pervasive lack of micronutrient-dense food security.

These findings strongly suggest that the current strategy of relying on individual dietary modification or sporadic supplementation is inadequate for high-risk regions. To effectively reduce the high incidence of NTDs in this region, policymakers must transition toward mandatory, large-scale food fortification programs as a primary public health intervention. Addressing this silent micronutrient emergency is not merely a clinical priority but a fundamental prerequisite for safeguarding maternal health and reducing the preventable burden of birth defects in Northern Ethiopia.

### Recommendations

Based on the findings of this study, the following actions are recommended to address the widespread folate deficiency among first-trimester pregnant women in the Tigray region: **Implement Mandatory Food Fortification:** The Ethiopian government should expedite the adoption and enforcement of national legislation mandating folic acid fortification of staple foods such as wheat and maize flour. This evidence-based, cost-effective intervention has been shown to significantly reduce the incidence of neural tube defects at the population level.

**Scale Up Preconception and Antenatal Supplementation:** Health authorities should expand access to periconceptional and early antenatal folic acid supplementation, with a particular focus on rural and underserved communities where utilization of health services remains limited. Integrating supplementation into routine maternal and reproductive health services is critical for timely coverage.

**Promote Dietary Diversification:** Community-based nutrition education programs should emphasize the importance of consuming folate-rich foods such as dark green leafy vegetables, legumes, eggs, and other animal-source foods. Local agricultural and food systems should be strengthened to enhance the availability, accessibility, and affordability of these nutrient-dense foods.

**Strengthen Family Planning and Reproductive Health Services:** In light of the high prevalence of unplanned pregnancies, it is essential to improve access to family planning services and reproductive health education. Empowering women to plan their pregnancies allows for better nutritional preparation during the critical preconception period.

**Provide Targeted Support for Rural Populations:** Tailored interventions should address the specific challenges faced by rural women, including limited health literacy, poor access to healthcare, and economic constraints. Strategies such as deploying mobile health units and leveraging community health workers can help bridge service gaps and deliver targeted nutrition support.

**Enhance Research and Surveillance:** Continued monitoring of folate status among women of reproductive age is vital for tracking progress and guiding interventions. Further research should explore other coexisting micronutrient deficiencies and evaluate the effectiveness of food-based, educational, and policy-level interventions.

### Limitation of the study

This study is strengthened by its use of red blood cell (RBC) folate as a biomarker a reliable and stable indicator of long-term folate status and by its relatively large, multisite hospital-based sample. However, several limitations should be considered when interpreting the findings. The facility-based design may limit generalizability to pregnant women who do not access antenatal care. Inflammatory markers were not measured, although they are not known to significantly influence RBC folate levels.

This study has several methodological limitations. First, only 5 of the 400 participants had RBC folate concentrations ≥400 ng/mL, resulting in substantial class imbalance. Although penalized Ridge regression and upsampling were used to explore potential predictors of folate sufficiency, upsampling does not generate new information and may amplify coefficient estimates by repeatedly sampling the same minority-class observations. Therefore, the regression findings should be interpreted as exploratory, and the study’s primary conclusions are based on the robust descriptive prevalence estimates. Finally, the cross-sectional study design precludes causal inference between dietary diversity and RBC folate status.

## References

[1] Naninck EFG, Stijger PC, Brouwer-Brolsma EM. The Importance of Maternal Folate Status for Brain Development and Function of Offspring. Adv Nutr. 2019;10(3):502–19. doi: 10.1093/advances/nmy120 31093652 PMC6520042

[2] Greenberg JA, Bell SJ, Guan Y, Yu Y-H. Folic Acid supplementation and pregnancy: more than just neural tube defect prevention. Rev Obstet Gynecol. 2011;4(2):52–9. doi: 10.18370/2309-4117.2017.34.57-63 22102928 PMC3218540

[3] Copp AJ, Stanier P, Greene NDE. Genetic Basis of Neural Tube Defects. Textbook of Pediatric Neurosurgery. Springer International Publishing. 2020. p. 2275–94. doi: 10.1007/978-3-319-72168-2_105

[4] Petersen JM, Parker SE, Benedum CM, Mitchell AA, Tinker SC, Werler MM. Periconceptional folic acid and risk for neural tube defects among higher risk pregnancies. Birth Defects Res. 2019;111(19):1501–12. doi: 10.1002/bdr2.1579 31433116 PMC7186568

[5] Zaganjor I, Sekkarie A, Tsang BL, Williams J, Razzaghi H, Mulinare J, et al. Describing the Prevalence of Neural Tube Defects Worldwide: A Systematic Literature Review. PLoS One. 2016;11(4):e0151586. doi: 10.1371/journal.pone.0151586 27064786 PMC4827875

[6] Blencowe H, Kancherla V, Moorthie S, Darlison MW, Modell B. Estimates of global and regional prevalence of neural tube defects for 2015: a systematic analysis. Ann N Y Acad Sci. 2018;1414(1):31–46. doi: 10.1111/nyas.13548 29363759

[7] Chen M-Y, Rose CE, Qi YP, Williams JL, Yeung LF, Berry RJ, et al. Defining the plasma folate concentration associated with the red blood cell folate concentration threshold for optimal neural tube defects prevention: a population-based, randomized trial of folic acid supplementation. Am J Clin Nutr. 2019;109(5):1452–61. doi: 10.1093/ajcn/nqz027 31005964 PMC7099800

[8] World Health Organization. Serum and red blood cell folate concentrations for assessing folate status in populations. Geneva: World Health Organization. 2015.

[9] Cordero AM, Crider KS, Rogers LM, Cannon MJ, Berry RJ. Optimal serum and red blood cell folate concentrations in women of reproductive age for prevention of neural tube defects: World Health Organization guidelines. MMWR Morb Mortal Wkly Rep. 2015;64(15):421–3. 25905896 PMC5779552

[10] Bechoff A, de Bruyn J, Alpha A, Wieringa F, Greffeuille V. Exploring the Complementarity of Fortification and Dietary Diversification to Combat Micronutrient Deficiencies: A Scoping Review. Curr Dev Nutr. 2023;7(2):100033. doi: 10.1016/j.cdnut.2023.100033 37180084 PMC10111601

[11] Ghotme KA, Arynchyna-Smith A, Maleknia P, Kancherla V, Pachon H, J Van der Wees P, et al. Barriers and facilitators to the implementation of mandatory folate fortification as an evidence-based policy to prevent neural tube defects. Childs Nerv Syst. 2023;39(7):1805–12. doi: 10.1007/s00381-023-05944-x 37209199 PMC10290612

[12] Darnton-Hill I. Public Health Aspects in the Prevention and Control of Vitamin Deficiencies. Curr Dev Nutr. 2019;3(9):nzz075. doi: 10.1093/cdn/nzz075 31598578 PMC6775441

[13] Zegeye AF, Mekonen EG, Tamir TT, Workneh BS. Prevalence and determinants of inadequate dietary diversity among pregnant women in four Sub-Saharan Africa countries: a multilevel analysis of recent demographic and health surveys from 2021 to 2022. Front Nutr. 2024;11:1405102. doi: 10.3389/fnut.2024.1405102 39301417 PMC11410580

[14] Soriano JM, Morales-Suarez-Varela M. Malnutrition during pregnancy and childhood: Ethiopian perspectives. Handbook of Public Health Nutrition: International, National, and Regional Perspectives. Cham: Springer Nature Switzerland. 2025. p. 1–28.

[15] Seid A, Dugassa Fufa D, Weldeyohannes M, Tadesse Z, Fenta SL, Bitew ZW, et al. Inadequate dietary diversity during pregnancy increases the risk of maternal anemia and low birth weight in Africa: A systematic review and meta-analysis. Food Sci Nutr. 2023;11(7):3706–17. doi: 10.1002/fsn3.3388 37457158 PMC10345738

[16] Zerfu TA, Biadgilign S. Pregnant mothers have limited knowledge and poor dietary diversity practices, but favorable attitude towards nutritional recommendations in rural Ethiopia: evidence from community-based study. BMC Nutr. 2018;4:43. doi: 10.1186/s40795-018-0251-x 32153904 PMC7050941

[17] Tesfay N, Hailu G, Habtetsion M, Woldeyohannes F. Birth prevalence and risk factors of neural tube defects in Ethiopia: a systematic review and meta-analysis. BMJ Open. 2023;13(11):e077685. doi: 10.1136/bmjopen-2023-077685 37940152 PMC10632862

[18] Berihu BA, Welderufael AL, Berhe Y, Magana T, Mulugeta A, Asfaw S, et al. High burden of neural tube defects in Tigray, Northern Ethiopia: Hospital-based study. PLoS One. 2018;13(11):e0206212. doi: 10.1371/journal.pone.0206212 30427877 PMC6235279

[19] Mekonen HK, Berhe Y, Berihu BA, Teka H, Hadgu A, Gebregziabher L, et al. A silent epidemic of major congenital malformations in Tigray, northern Ethiopia: hospital-based study. Sci Rep. 2021;11(1):21035. doi: 10.1038/s41598-021-00240-7 34702882 PMC8548534

[20] Berihu BA, Mulugeta A, Magana T, Tessema M, Gebreegziabher T, Berhe Y, et al. Neural tube defects in a war-torn Tigray regional state of Ethiopia: a retrospective study of 54,626 deliveries. BMC Pregnancy Childbirth. 2025;25(1):108. doi: 10.1186/s12884-025-07254-3 39901097 PMC11789397

[21] United Nations Children’s Fund UNICEF. Tigray region briefing note. Addis Ababa: UNICEF. 2015.

[22] Central Statistical Agency (CSA) [Ethiopia], ICF International. Ethiopia Demographic and Health Survey 2011. Addis Ababa, Ethiopia, and Calverton (MD): CSA and ICF International. 2012.

[23] Central Statistical Agency (CSA) [Ethiopia], I C F. Ethiopia Demographic and Health Survey 2016: Key Indicators Report. Addis Ababa, Ethiopia, and Rockville (MD): CSA and ICF. 2016.

[24] Food and Agriculture Organization of the United Nations. Minimum dietary diversity for women: A guide for measurement. Rome: FAO; 2016.

[25] Tefera AA, Seifu D, Menon M, Talargia F, Belete AM. Red blood cell folate level and associated factors of folate insufficiency among pregnant women attending antenatal care during their first trimester of pregnancy in Addis Ababa, Ethiopia. SAGE Open Med. 2022;10:20503121221118987. doi: 10.1177/20503121221118987 36051782 PMC9424885

[26] Sisay BG, Tamirat H, Sandalinas F, Joy EJM, Zerfu D, Belay A, et al. Folate Deficiency Is Spatially Dependent and Associated with Local Farming Systems among Women in Ethiopia. Curr Dev Nutr. 2022;6(5):nzac088. doi: 10.1093/cdn/nzac088 35669042 PMC9154233

[27] Gebremichael B, Roba HS, Getachew A, Tesfaye D, Asmerom H. Folate deficiency among women of reproductive age in Ethiopia: A systematic review and meta-analysis. PLoS One. 2023;18(5):e0285281. doi: 10.1371/journal.pone.0285281 37155667 PMC10166565

[28] Ethiopian Public Health Institute, Ministry of Health. Ethiopian National Micronutrient Survey Report. Addis Ababa, Ethiopia: Ethiopian Public Health Institute. 2016.

[29] Mamme NY, Roba HS, Fite MB, Asefa G, Abrahim J, Yuya M, et al. Serum folate deficiency and associated factors among pregnant women in Haramaya District, Eastern Ethiopia: a community-based study. BMJ Open. 2023;13(5):e068076. doi: 10.1136/bmjopen-2022-068076 37156586 PMC10174039

[30] Rohner F, Northrop-Clewes C, Tschannen AB, Bosso PE, Kouassi-Gohou V, Erhardt JG, et al. Prevalence and public health relevance of micronutrient deficiencies and undernutrition in pre-school children and women of reproductive age in Côte d’Ivoire, West Africa. Public Health Nutr. 2014;17(9):2016–28. doi: 10.1017/S136898001300222X 24171836 PMC11107373

[31] Ndiaye NF, Idohou-Dossou N, Diouf A, Guiro AT, Wade S. Folate Deficiency and Anemia Among Women of Reproductive Age (15-49 Years) in Senegal: Results of a National Cross-Sectional Survey. Food Nutr Bull. 2018;39(1):65–74. doi: 10.1177/0379572117739063 29129112

[32] Ouédraogo S, Koura GK, Accrombessi MMK, Bodeau-Livinec F, Massougbodji A, Cot M. Maternal anemia at first antenatal visit: prevalence and risk factors in a malaria-endemic area in Benin. Am J Trop Med Hyg. 2012;87(3):418–24. doi: 10.4269/ajtmh.2012.11-0706 22826498 PMC3435342

[33] Vanderjagt DJ, Brock HS, Melah GS, El-Nafaty AU, Crossey MJ, Glew RH. Nutritional factors associated with anaemia in pregnant women in northern Nigeria. J Health Popul Nutr. 2007;25(1):75–81. 17615906 PMC3013266

[34] Haidar J. Prevalence of anaemia, deficiencies of iron and folic acid and their determinants in Ethiopian women. J Health Popul Nutr. 2010;28(4):359–68. doi: 10.3329/jhpn.v28i4.6042 20824979 PMC2965327

[35] Haidar J, Melaku U, Pobocik RS. Folate deficiency in women of reproductive age in nine administrative regions of Ethiopia: An emerging public health problem. S Afr J Clin Nutr. 2010;23:132–7.

[36] Modjadji SE, Alberts M, Mamabolo RL. Folate and iron status of South African non-pregnant rural women of childbearing age before and after food fortification. S Afr J Clin Nutr. 2007;20:89–93.

[37] Shahab‐Ferdows S, Engle‐Stone R, Hampel D, Ndjebayi A, Nankap M, Brown K, et al. Risk factors for folate deficiency differ from those for vitamin B12 deficiency in Cameroonian women and children (119.7). The FASEB Journal. 2014;28(S1). doi: 10.1096/fasebj.28.1_supplement.119.7 26446486

[38] John SE, Azizi K, Hancy A, Twin’omujuni A, Katana D, Shine J, et al. The prevalence and risk factors associated with Iron, vitamin B12 and folate deficiencies in pregnant women: A cross-sectional study in Mbeya, Tanzania. PLOS Glob Public Health. 2023;3(4):e0001828. doi: 10.1371/journal.pgph.0001828 37083656 PMC10121015

[39] Rogers LM, Cordero AM, Pfeiffer CM, Hausman DB, Tsang BL, De-Regil LM, et al. Global folate status in women of reproductive age: a systematic review with emphasis on methodological issues. Ann N Y Acad Sci. 2018;1431(1):35–57. doi: 10.1111/nyas.13963 30239016 PMC6282622

[40] García-Casal MN, Osorio C, Landaeta M, Leets I, Matus P, Fazzino F, et al. High prevalence of folic acid and vitamin B12 deficiencies in infants, children, adolescents and pregnant women in Venezuela. Eur J Clin Nutr. 2005;59(9):1064–70. doi: 10.1038/sj.ejcn.1602212 16015269

[41] Karaoglu L, Pehlivan E, Egri M, Deprem C, Gunes G, Genc MF, et al. The prevalence of nutritional anemia in pregnancy in an east Anatolian province, Turkey. BMC Public Health. 2010;10:329. doi: 10.1186/1471-2458-10-329 20537176 PMC2904273

[42] Quinn M, Halsey J, Sherliker P, Pan H, Chen Z, Bennett DA, et al. Global heterogeneity in folic acid fortification policies and implications for prevention of neural tube defects and stroke: a systematic review. EClinicalMedicine. 2023;67:102366. doi: 10.1016/j.eclinm.2023.102366 38169713 PMC10758734

